# Configurational Entropy Components and Their Contribution to Biomolecular Complex Formation

**DOI:** 10.1021/acs.jctc.8b01254

**Published:** 2019-05-01

**Authors:** Markus Fleck, Bojan Zagrovic

**Affiliations:** † University of Vienna, Max F. Perutz Laboratories, Department of Structural and Computational Biology, Campus Vienna Biocenter 5, Vienna 1030, Austria; ‡ University of Vienna, Faculty of Chemistry, Department of Computational Biological Chemistry, Währinger Straße 17, Vienna 1090, Austria

## Abstract

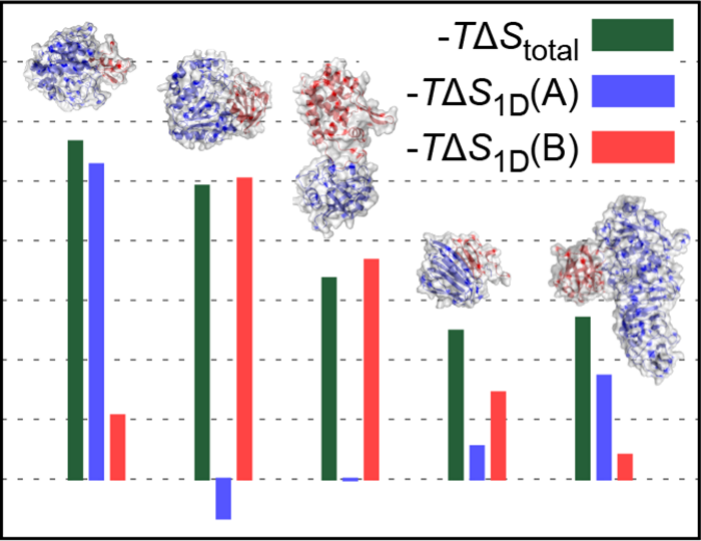

Configurational entropy change is a central constituent of the free energy change in noncovalent interactions between biomolecules. Due to both experimental and computational limitations, however, the impact of individual contributions to configurational entropy change remains underexplored. Here, we develop a novel, fully analytical framework to dissect the configurational entropy change of binding into contributions coming from molecular internal and external degrees of freedom. Importantly, this framework accounts for all coupled and uncoupled contributions in the absence of an external field. We employ our parallel implementation of the maximum information spanning tree algorithm to provide a comprehensive numerical analysis of the importance of the individual contributions to configurational entropy change on an extensive set of molecular dynamics simulations of protein binding processes. Contrary to commonly accepted assumptions, we show that different coupling terms contribute significantly to the overall configurational entropy change. Finally, while the magnitude of individual terms may be largely unpredictable a priori, the total configurational entropy change can be well approximated by rescaling the sum of uncoupled contributions from internal degrees of freedom only, providing support for NMR-based approaches for configurational entropy change estimation.

## 1 Introduction

Noncovalent interactions between macromolecules are fundamental to a large number of biological processes including transcription, translation, cell signaling, and many other.^1^ Given an isothermal–isobaric ensemble with a constant number of particles (*NPT*), the Gibbs free energy change [see, e.g., ref (2)]
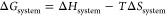
1captures the likelihood for such a binding process to occur, together with the equilibrium fractions of the species involved. Importantly, the entropic term (−*T*Δ*S*_system_) remains largely underexplored when it comes to biological macromolecules. This especially concerns the configurational entropy part of the total entropy change, which stems from the solute degrees of freedom only and is notoriously difficult to measure experimentally^3−5^ or calculate from atomistic simulations.^6−18^ It has traditionally been assumed that the configurational entropy change is negligible in comparison to the change in solvent entropy.^19^ Recently, however, it was experimentally demonstrated that the configurational entropy contribution in the case of proteins can be of similar magnitude as the solvent entropy contribution^3−5^ and can thus potentially have a strong impact on the thermodynamics of protein interactions. In a more applied context, deeper insight into configurational entropy and the basic physical principles that govern its response to changes in biomolecular dynamics could significantly improve computational drug design by helping to overcome enthalpy/entropy compensation.^20−22^ In the present work, we analyze the individual contributions to configurational entropy change of protein binding, stemming from internal^12,23−28^ and external (rigid body rototranslational) degrees of freedom. Going beyond the previous studies of entropy change in protein–protein interactions, such as those involving normal mode calculations,^29,30^ we investigate here the importance and the relative magnitude of the often ignored coupling (correlation) terms between internal and external degrees of freedom in BAT coordinates. Following previous work,^31^ we employ an entropy decomposition^32^ known as the mutual information expansion (MIE) in its analytical form. There exists a well-developed theoretical apparatus for the decomposition of such coupling terms (see, e.g., refs (12) and (33) and references therein), mainly for liquids. However, numerical application of MIE as an approximation of the configurational entropy is rather novel in the case of configurational entropy of biomolecules.^12^

In the pioneering work by Gilson and co-workers,^12^ the MIE expansion is taken at the level of single degrees of freedom as opposed to sets of degrees of freedom, which, as mentioned above, will be treated in this work. The obtained numerical coupling terms can then be summed up to approximate the analytical MIE coupling terms at the level of the sets of degrees of freedom, e.g., external with internal or main-chain with side-chain. In this context, Gilson and co-workers^34^ have recently discussed and reviewed the general significance of coupling corrections in configurational entropy estimation. Importantly, they have applied a more recently developed variant of the MIE approximation, the maximum information spanning tree (MIST) approximation.^10,35^ However, MIST/MIE analysis of the coupling terms in proteins resulting from the partitioning into external and internal degrees of freedom is, to the best of our knowledge, limited to a single case study,^11^ employing the MIE approximation at pairwise order.

**Figure 1:**
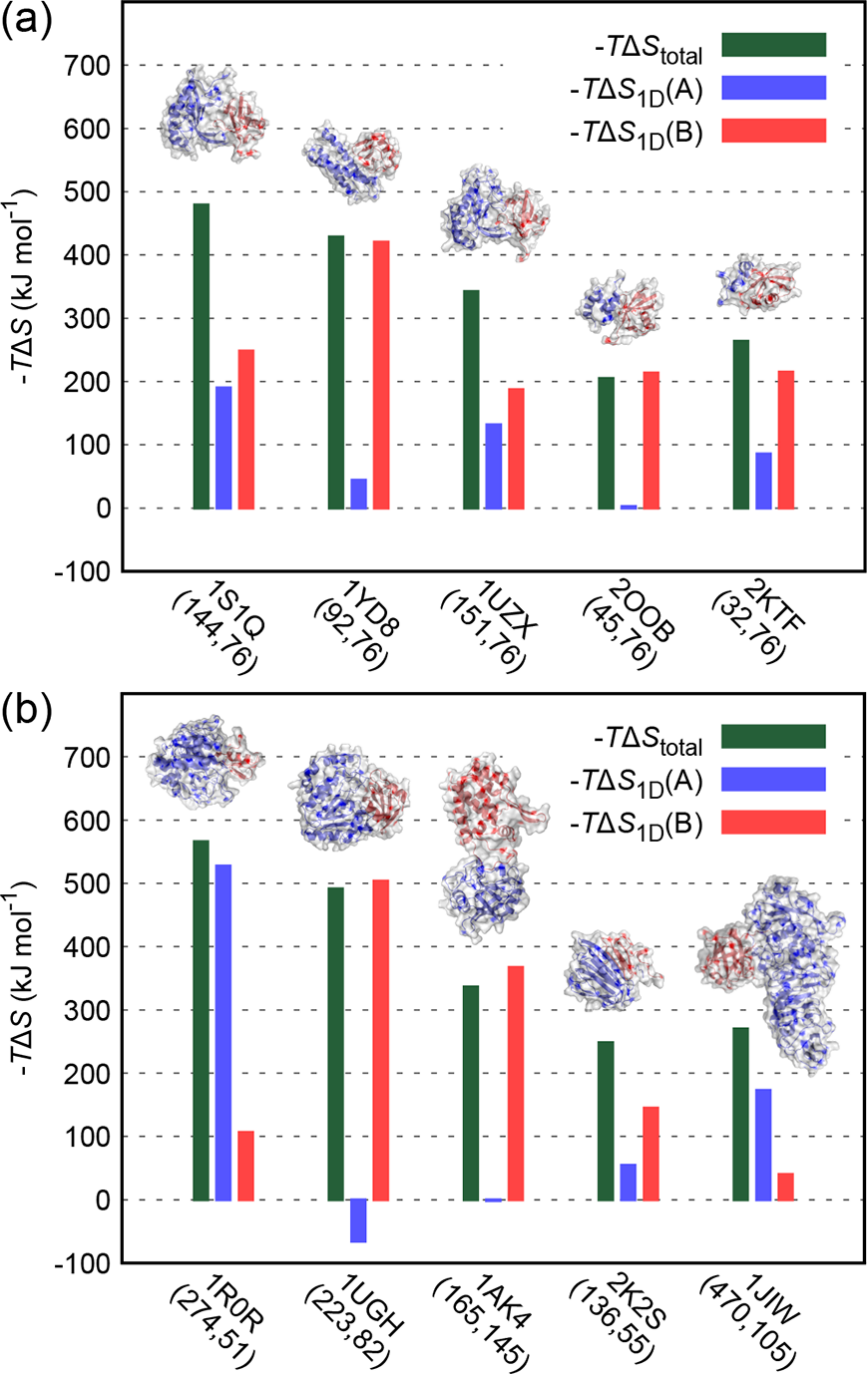
Structures of the simulated protein complexes together with the computed configurational entropy contributions to the Gibb’s free energy change of binding of the whole complexes (−*T*Δ*S*_total_) or binding partners alone [−*T*Δ*S*_1D_(A) and – *T*Δ*S*_1D_(B)]. The latter values stem from the internal degrees of freedom of individual binding partners without any coupling (mutual information) contributions included. On the *x*-axis, we give the PDB code^41,42^ of each complex together with the number of amino acids in each partner (in parentheses). In (a), the second binding partner B (colored red) is always ubiquitin. Please see Table 1 for further details concerning the simulated complexes.

Here, by distilling the previous work^31,32^ to a compact form, we arrive at a decomposition of entropy into sets of external and internal degrees of freedom in the form of a general framework, which takes advantage of separable terms in the underlying potential energy. We compute these contributions to configurational entropy change at pairwise order for a large set of typical protein complexes (Figure 1 and Table 1). The isolated binding partners and their binary complexes are captured using microsecond-level classical molecular dynamics simulations (for methodological details and a full description of the simulated set, please see refs (36−38)). The simulated set exhibits a wide range of physical sizes and secondary- and tertiary-structure classes of individual binding partners as well as a variety of the total configurational entropy changes and the uncoupled configurational entropy changes of individual binding partners. Note that five of the simulated complexes involve ubiquitin as one of the binding partners (Figure 1a), a well-folded, biologically important protein frequently used in biophysical studies; we highlight these complexes for clarity in all of our analyses. This large-scale investigation is made possible by our recent parallel implementation^36^ of MIE/MIST. For three representative complexes, we carry out an extensive analysis of configurational entropy convergence, leading to several notable results. Additionally, as there exists a certain fundamental arbitrariness^31,39,40^ in decomposing the entropy over external and internal degrees of freedom, we provide an analysis of the impact of different decomposition choices (Figure 5). We demonstrate that several coupling terms contribute significantly to the overall configurational entropy change across different proteins, contrary to commonly accepted assumptions. Finally, we provide a justification for the experimental estimation of the total entropy change from the leading internal uncoupled entropy terms even under these circumstances.

**Table 1:** Simulated Protein Set

name	# atoms^a^	PDB code^b^	complex^c^	–*T*Δ*S*_1D_^d^
Tsg101 protein	1480	1KPP	1S1Q	190.0
ubiquitin	760	1UBQ	1S1Q	248.3
gGGA3 Gat domain^e^	949	1YD8*	1YD8	44.0
ubiquitin	760	1UBQ	1YD8	420.4
ESCRT-I complex subunit VPS23	1493	3R3Q	1UZX	131.6
ubiquitin	760	1UBQ	1UZX	187.5
E3 ubiquitin–protein ligase CBL-B	457	2OOA	2OOB	2.4
ubiquitin	760	1UBQ	2OOB	213.5
polymerase iota ubiquitin-binding motif	457	2L0G	2KTF	85.5
ubiquitin	760	1UBQ	2KTF	215.1
subtilisin Carlsberg	2433	1SCD	1R0R	527.4
ovomucoid	498	2GKR	1R0R	106.6
uracil–DNA glycosylase	2333	1AKZ	1UGH	–65.7
uracil–DNA glycosylase inhibitor	788	1UGI	1UGH	503.8
PPIase A	1641	1W8V	1AK4	–1.4
PR160Gag-Pol	1408	2PXR	1AK4	367.4
micronemal protein 1	1226	2BVB	2K2S	54.5
micronemal protein 6	496	2K2T	2K2S	145.0
alkaline protease	4503	1AKL	1JIW	173.1
alkaline protease inhibitor	997	2RN4	1JIW	40.0

a Number of force field atoms of individual proteins.

b PDB codes^41,42^ of individual proteins.

c PDB codes of complexes.

d Entropy change from internal degrees of freedom of individual binding partners upon complex formation without any coupling (mutual information) contributions, given in kJ mol^–1^, as in Figure 1.

e The constituent GGA3 Gat domain was extracted from the PDB structure of the 1YD8 complex and named 1YD8* accordingly.

## 2 Theory

### 2.1 Configurational Entropy of the Binding Process

An expression for the configurational entropy of a single molecule or complex can be derived from the quasi-classical entropy integral^43,44^
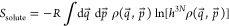
2where *R* represents the universal gas constant, *h* the Planck constant, *N* the number of atoms in the molecule, and ρ the classical phase-space probability density function (pdf). *q⃗* and *p⃗* denote, respectively, the spatial degrees of freedom and the canonically conjugate momenta in Cartesian coordinates. Note that due to the factor *h*^3*N*^ this integral cannot be split into momentum and spatial parts while preserving physically correct dimensions for both quantities.^38,43^ As in this work we are concerned with the spatial part of the entropy (labeled just *S* to simplify the notation), a convenient choice for separating off the momentum entropy *S*_m_ is
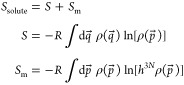
3The momentum entropy then evaluates to^38,43^
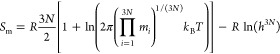
4Here, *m*_*i*_ denotes the mass vector of the solute, and *k*_B_ is the Boltzmann constant. Note that this expression is constant if the temperature and atomic composition remain fixed. Under the assumption of a vanishing external field and a concentration *C*° associated with a container of volume *V*° = 1/*C*° for a single molecule (or complex), the spatial part of eq 2 can be evaluated to^11,12,43^
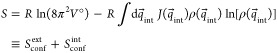
5where *J*(*q⃗*_int_) denotes the Jacobian of the chosen molecule internal coordinates (such as anchored Cartesian^23,24^ or BAT^12,23−28^ coordinates). Thus, the second term on the right-hand side captures the integration over the chosen 3*N* – 6 internal degrees of freedom *q⃗*_int_. The term *R* ln(8π^2^*V*°) in eq 5 results from integration over the 6 external degrees of freedom, which in the absence of an external field can be carried out analytically.^26^ Here, following a common practice, we choose a standard concentration of *C*° = 1/*V*° = 1 mol L^–3^. As this term as well as the momentum entropy at fixed temperature and atomic composition is constant,^38,43^ the second term on the right-hand side of eq 5 alone is often referred to as configurational entropy. Therefore, for a single molecule, sampling the internal, spatial pdfs is sufficient to calculate the total entropy contribution to the Gibbs free energy change from the solute. Importantly, while neither the momentum entropy (eq 4) nor the spatial entropy (eq 5), as mentioned above, exhibit physically correct dimensions, the problematic terms cancel for entropy differences.^38,43^ Thus, differences (and differences only) of these quantities bear physically valid dimensions of entropy.

We now begin our analysis of the configurational entropy change of a binary binding process by deriving the configurational entropy of the unbound state (including external degrees of freedom), followed by the derivation of the configurational entropy of the bound state. As mentioned in the introduction, both derivations follow a previously discussed strategy,^31^ making extensive use of the analytical MIE.^32^ The configurational entropy change upon binding is then obtained by subtraction. As no other approximations are made apart from the assumption of a vanishing external field, the end result as well as all intermediate results are analytically exact in the classical limit.

### 2.2 Configurational Entropy of the Unbound State from a Statistical-Mechanical Perspective

In the unbound state, the molecules are assumed to be infinitely far apart, and we assume no external fields. The notation used for describing different terms is given in Table 2. Note that from now on we drop the vector symbol for a more convenient notation. With these assumptions and notation, the potential energy separates as
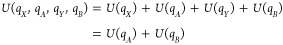
6Here, without a loss of generality, the external potential constants are set to zero. Analogously, the pdf factorizes into
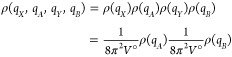
7The factor 8π^2^*V*° results from the homogeneous probability distribution with respect to the position in the container volume, the full solid angle, as well as the external torsional degree of freedom of the molecules. Finally, using the corresponding external entropy terms *R* ln(8π^2^*V*°) from eq 5 and the notation of Table 2, the spatial entropy of the unbound state is given as
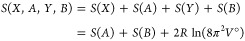
8

**Table 2:** Nomenclature of the Degrees of Freedom

*q*_*X*_	external degrees of freedom molecule 1
*q*_*Y*_	external degrees of freedom molecule 2^a^
*q*_*A*_	internal degrees of freedom molecule 1
*q*_*B*_	internal degrees of freedom molecule 2
*X*	random variables from *q*_*X*_
*Y*	random variables from *q*_*Y*_
*A*	random variables from *q*_*A*_
*B*	random variables from *q*_*B*_
∼	a quantity associated with the bound state
*S*_1D_	entropy from marginal 1D probability density functions only (within a given subsystem)
*I*_2D_	mutual information of 2D and higher probability density functions (within a given subsystem^b^)
*I*_2_	pairwise mutual information of 2D and higher probability density functions (shared between two subsystems^b^)
*I*_3_	triplet mutual information of 3D and higher probability density functions (shared between three subsystems^c^)

a In the reference frame of molecule 1.

b Numerically approximated by 2D probability density functions in this work.

c Not treated numerically in this work.

### 2.3 Configurational Entropy of the Unbound State from an Information-Theoretic Perspective

The same result from the previous subsection can be derived in an information-theoretic framework. First, the MIE is denoted as^32^
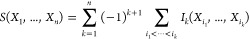
9where
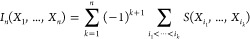
10are the so-called higher-order mutual information (MI) terms of order *n*. Note however that *I*_1_(*X*_*i*_) = *S*(*X*_*i*_). For further derivation, it will be convenient to first prove the following summary of the previous work^32^ in a general form
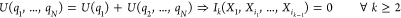
11Here, for every set of degrees of freedom *q*_*i*_, *X*_*i*_ denotes the corresponding random variable. This equation states that, if a given set of degrees of freedom can be separated out in the energy function, all (higher-order) MI terms describing the coupling of this set to any possible subset (including the full set) of the remaining degrees of freedom vanishes simultaneously. The proof then proceeds as follows. Analogously to eqs 6–8, we have
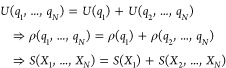
12Then, from eq 10, it follows for the pairwise MI
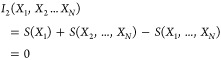
13As for pairwise MI, we have^45^
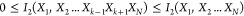
14All pairwise combinations involving *X*_1_ vanish simultaneously. Furthermore, all higher-order MI terms can be expanded recursively as a sum of such vanishing pairwise MI combinations with *X*_1_ using^32^
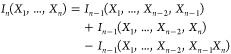
15This completes the proof of eq 11. Now, applying the MIE (eq 9) for the subsystems treated in this work yields the expansion
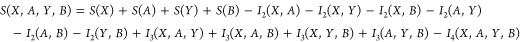
16As, according to eq 6, we have *U*(*q*_*X*_,*q*_*A*_,*q*_*Y*_,*q*_*B*_) = *U*(*q*_*X*_) + *U*(*q*_*A*_) + *U*(*q*_*Y*_) + *U*(*q*_*B*_), all MI terms vanish for the unbound state. Then, in accordance with eq 8, one obtains
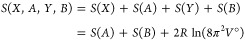
17

### 2.4 Configurational Entropy of the Bound State

In the bound state, the two molecules are close together, and therefore, the orientation of molecule 2 with respect to molecule 1 contributes to the potential energy. Because in the bound state the internal degrees of freedom between the molecules also influence each other, only the external degrees of freedom of the first molecule remain separable (as they anchor the whole complex and we assume no external field). Thus, denoting the degrees of freedom in the bound state with a tilde, one can write
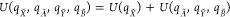
18Then, using eq 11 for the vanishing MI terms and the MIE for the present subsystems (see eq 16), together with the fact that as before *S*(*X̃*) = *R* ln(8π^2^*V*°), the configurational entropy in the bound state can be written as
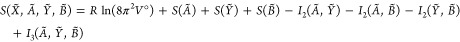
19Note that the same result could be derived by using *S*(*X̃*,*Ã*,*Ỹ*,*B̃*) = *S*(*X̃*) + *S*(*Ã*,*Ỹ*,*B̃*) from the statistical mechanical framework due to eq 18 and then applying the MIE (eq 9) just to *S*(*Ã*,*Ỹ*,*B̃*).

### 2.5 Configurational entropy change upon binding

Using the results from the previous subsections, the configurational entropy change upon binding can be obtained by subtracting eq 17 (or equivalently eq 8) from eq 19 as
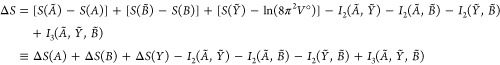
20This final result describes the fully analytical configurational entropy change in the absence of an external field expressed in terms of contributions from external and internal degrees of freedom of the molecules involved. It follows from the singular assumption of the form of the potential energy function in eqs 6 and 18 in the classical limit without any further approximations. For the external degrees of freedom of the second molecule, the term Δ*S*(*Y*) = *S*(*Ỹ*) – ln(8π^2^*V*°) expresses the rototranslational restriction upon binding to the first molecule, in contrast to the motional freedom in the unbound state. Note also that the only contributions that reflect the coupling between the four subsystems stem from the MI in the bound state.

## 3 Results and Discussion

Using the above analytical framework, we analyzed the relative importance of the individual contributions to configurational entropy change in the case of 10 protein complexes shown in Figure 1. As a consequence of the limitations of in silico sampling, application of the MIST approximation at an order higher than pairwise is currently not possible for proteins of biologically relevant sizes. However, one can further dissect eq 20 by separating the uncoupled configurational entropy from the mutual information terms within a given subsystem. Here, note that while, e.g., *S*(*A*) appears as a one-dimensional term at the level of individual subsystems, when it comes to degrees of freedom, it stems from a high-dimensional probability density function, which one can expand via eq 9. The same holds for *I*_2_ terms: while at the level of individual subsystems they appear as pairwise mutual information terms, at the level of degrees of freedom they are described by higher-order mutual information terms as in eq 9. Separating off the coupling terms within one subsystem leads then to
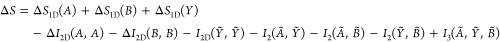
21Here, as denoted in Table 2, *S*_1D_ refers to the sum of the *I*_1_ terms in eq 9 and *I*_2*D*_ to the sum of all terms *I*_*k*_ with *k* > 1, both referring to the equation expressed at the level of degrees of freedom and within one subsystem. While our analysis approximates all *I*_2D_ and *I*_2_ terms in eq 21 from 2D pdfs over the degrees of freedom, the triplet term *I*_3_ inherently has dimensions ≥ 3 and is, thus, difficult to sample properly. Note that the term *I*_2D_(*Ỹ*,*Ỹ*) is zero for the unbound state, corresponding to the total motional freedom of the molecules. Thus, *I*_2D_(*Ỹ*,*Ỹ*) enters the equation directly without making a difference in the case of the unbound state.

### 3.1 Convergence Analysis

Before analyzing and comparing individual contributions to the configurational entropy change, we would first like to discuss the convergence of our computational estimates. The uncertainty in configurational entropy calculations stems, in principle, from two main sources. First, the underlying simulations need to accurately and exhaustively sample the configurational space explored by a given molecule. While the question of force field accuracy is an important one, its adequate treatment is beyond the scope of the present study. On the other hand, the question of how exhaustively the phase space is sampled may be addressed by monitoring the convergence of the configurational entropy change and its components as a function of simulated time. Second, uncertainty is also influenced by the intrinsic properties of different configurational entropy components and their dependence on sufficient sample size. This question may be addressed by analyzing samples of different size coming from a shuffled trajectory in which the ordering of individual snapshots is randomized, thus removing the physical sources of uncertainty. We have carried out both of these types of analysis for three representative complexes in our set: the smallest one (PDB code 2KTF), the largest one (PDB code 1JIW), and a medium-size one involving *S*_1D_ terms with noteworthy properties, as further discussed below (PDB code 1UGH).

**Figure 2:**
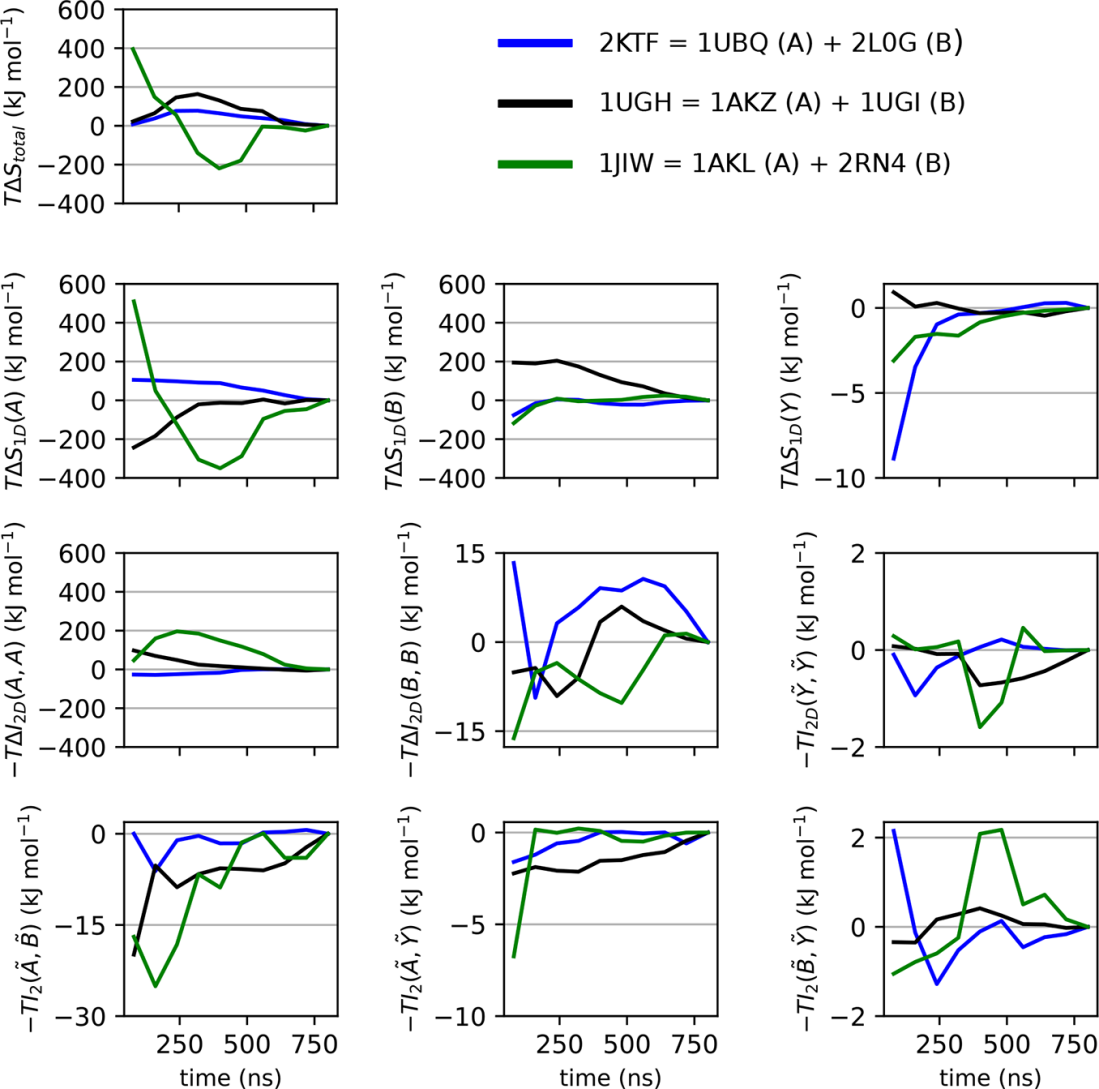
Dependence of the calculated configurational entropy change and its components on simulated time for three representative complexes. For each complex and contribution, we give values based on simulated samples with a 1 ps output frequency whose total size increases incrementally in steps of 80 ns. For visualization purposes, the curves have been shifted to converge to zero when reaching their final value at the total simulation length of 800 ns. The reference to binding partners A and B in different complexes as denoted by their PDB codes is the same as that in Figure 1 and Table 1.

When it comes to total entropy change and its convergence as a function of physical time, the complexes converge to within 9, 7, and 25 kJ/mol from the final value for the 2KTF, 1UGH, and 1JIW complexes, respectively, already 80 ns before the end of the simulated trajectories (Figure 2). Considering the configurational entropy components, for 1JIW, the principal determinant of convergence is the *T*Δ*S*_1D_ term of its larger protein 1AKZ, making up 4503 of its total 5500 atoms. The corresponding mutual information term −*T*Δ*I*_2D_, in fact, converges considerably better (Figure 2): for example, over the last 80 ns, *T*Δ*S*_1D_ rises by about 46 kJ mol^–1^, while −Δ*I*_2D_ drops by only 5 kJ mol^–1^. In fact, an analogues statement can be made for all six proteins analyzed: −*T*Δ*I*_2D_ does not constitute the limiting factor for convergence. It is rather the *T*Δ*S*_1D_ terms that are more problematic to converge. Another noteworthy observation can be made for 1UGH: the larger protein 1AKZ, constituting 2333 of the 3121 atoms, converges surprisingly well in all of its components (Figure 2). The *T*Δ*S*_1D_ terms of the smaller protein 1UGI, on the other hand, still drop by 35 kJ mol^–1^ over the last 160 ns. The reason for this is likely the magnitude of the respective *T*Δ*S*_1D_ values, which is −66 kJ mol^–1^ for 1AKZ and a considerable 504 kJ mol^–1^ for 1UGI after the full 800 ns of the simulations. This suggests that physical size is not necessarily the deciding factor in convergence.

The other terms considered in this study, *T*Δ*S*_1D_(*Y*), −*TI*_2D_(*Ỹ*,*Ỹ*), −*TI*_2_(*Ã*,*B̃*), −*TI*_2_(*Ã*,*Ỹ*), and −*TI*_2_(*B̃*,*Ỹ*), show rather satisfactory convergence properties for all complexes analyzed, with their values coming to within approximately 2, 2, 5, 2, and 2 kJ mol^–1^, respectively, of the final values already in a few 80 ns steps.

What is left to discuss are the convergence properties of 1UBQ in the 2KTF complex. While ubiquitin is a stable, well-folded protein, its configurational entropy converges rather slowly, especially in its *T*Δ*S*_1D_ terms, which still drop by about 27 kJ mol^–1^ over the last 160 ns. The reason for this likely stems from the fact that, while well-folded, ubiquitin explores different conformational substates on a time scale that is slow compared to the simulation length of 800 ns. Indeed, the excellent convergence of configurational entropy changes and their components for all three complexes in the analysis of shuffled trajectories, with all terms converging to within 6 kJ/mol or less from the final value already within the first 80 ns (Figure SI 1), strongly suggests that the key determinant for convergence is not the sheer number of frames used for the configurational entropy calculation, but rather the quality of the underlying coverage of the phase space. In fact, the initial convergence in the analysis of shuffled trajectories turned out to be so rapid for all six proteins of the three complexes studied that we had to fine-grain the first 80 ns to steps of 8 ns to produce SI Figure 1.

Putting the convergence issues aside, the final configurational entropy change values, as calculated here, may seem relatively high. There are three separate issues that need to be mentioned in this regard. First, the MIST approximation is by definition an upper bound on the absolute configurational entropy and, if the underlying absolute values are too high, it is likely that the corresponding differences will show the same trend. Note, however, that when compared to the values obtained by the quasi-harmonic approximation the MIST configurational entropy differences are actually lower by a factor of approximately 3.^38^ Next, a large change in entropy is frequently accompanied by a large change in enthalpy, resulting in a moderate value for the relevant free energy change.^3^ In this sense, our results could very much be physically meaningful. Finally, the main experimental estimates of configurational entropy changes in protein interactions are derived from the changes in the NMR methyl order parameters by using a linear relationship between the two.^38,46^ While the proteins in our set indeed exhibit somewhat higher values of configurational entropy change as compared to the proteins that have been studied experimentally,^46^ they also explore a significantly larger range of order parameter changes (a factor of ∼3). Taking this into the account, one could claim that our results are approximately consistent with the experimentally measured magnitude of configurational entropy change.

### 3.2 Evaluation of Contributions to Configurational Entropy Change

**Figure 3:**
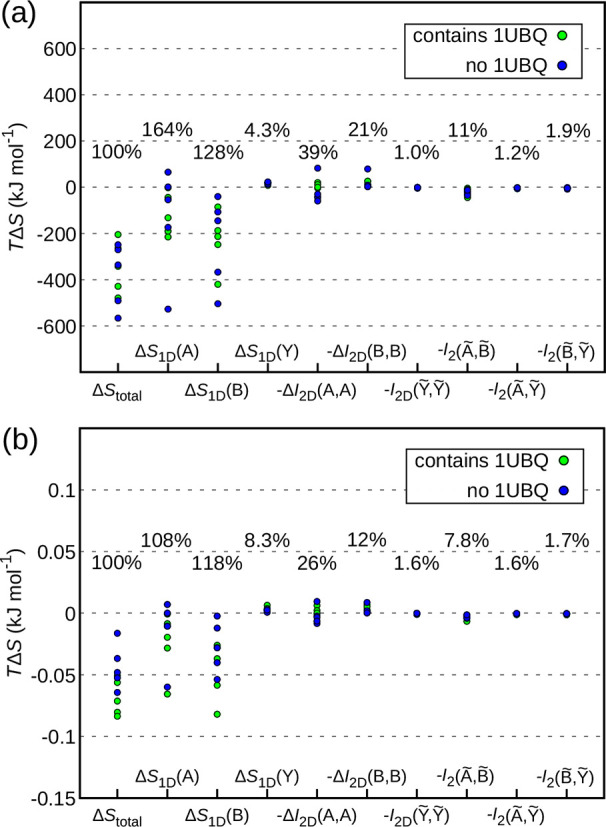
Contributions to configurational entropy change upon protein binding. Every column represents one of the contributions in eq 21, and every binding process contributes one data point in every column. The percentage values represent the span in the given column in relation to the span of the first column describing the total configurational entropy change. Green points denote complexes involving ubiquitin (Figure 1a), while blue points denote complexes not involving ubiquitin (Figure 1b). (b) Same as (a) but normalized by the number of degrees of freedom of the complex for each binding process, thus illustrating size dependence.

Acknowledging the uncertainties discussed in the previous section, we now turn back to the numerical investigation of eq 21. In Figure 3a, we evaluate this breakdown on our simulated set, each captured by one point in every column, as calculated from the pairwise order MIST approximation. The percentage values given in the graph capture the span of the values in reference to the span of the column Δ*S*_total_. Thus, these values can be interpreted as the numerical measure of the importance of a given contribution. We have opted for such a means of comparing different terms because taking the ratios between individual components for the same binding process, while seemingly more natural, results in some cases in misleadingly extreme values. As expected, the 1D terms of the internal degrees of freedom contribute the most, followed by the coupling within the molecules. The coupling between the internal degrees of freedom of the two molecules makes up 11% of the total variation. Note that, in absolute terms, this corresponds to a variation of 40 kJ mol^–1^. The smallest variation stems from arguably the most exotic term: the coupling of the external degrees of freedom of molecule 2 with respect to molecule 1 with themselves. However, although fractionally minor, this 1% percent of the span still makes up for 3.6 kJ mol^–1^, a value that could have physical and biological significance.

It is of interest, especially in the context of rational drug design, to assess whether the above results hold if smaller molecules are involved. To investigate this, we have analyzed the relationship between different configurational entropy contributions normalized by the number of degrees of freedom (3*N* – 6, where *N* is the total number of atoms) for each binding process (Figure 3b). This normalization down-weights the binding contributions of larger complexes or, i.e., up-weights those of the smaller complexes. For this reason, *Y*, which is comprised of a small but constant number of six external degrees of freedom regardless of the size of the complex, gains in importance. This is reflected in Δ*S*_1D_(*Y*) almost doubling. Also, the other terms involving *Y* tend to rather increase their impact [with the minor outlier −*I*_2D_(*B̃*,*Ỹ*)]. The fact that the smallest complex in this study is comprised of 1094 atoms together with the fact that the span of Δ*S*_1D_(*Y*) increases already by a factor of 2 demonstrates the importance of these external degrees of freedom as well as all of their couplings when investigating small systems. Thus, from an entropic point of view, retaining as much rotational and translational freedom as possible at the binding site should turn out especially beneficial for small ligands such as many drug compounds. However, due to the enthalpy/entropy compensation,^20−22^ one should also consider the impact on the enthalpic component of any practical optimization in this direction.

Note that there exists a fundamental arbitrariness in separating external from internal contributions, as already discussed by Gilson and co-workers.^31,39,40^ In the BAT coordinate system,^12,23−28^ this is reflected in the choice of root atoms from which the construction of the coordinate system is initiated. Accordingly, in the bound state, a nonphysical pseudobond is introduced connecting to the root atoms of the second molecule in order to form a complete coordinate system. For this reason, we numerically explore the impact of this largely arbitrary choice by performing our calculations for 5 different sets of root atoms in the second molecule for each of the 10 protein complexes. While Figure 3a illustrates the values chosen from the root atoms that minimize Δ*S*_1D_(*Y*), as proposed in ref (31), Figure 5 shows the changes of the values with respect to the maximization of such terms. The spans relative to the total entropy change in Figure 3a suggest that the global importance of the individual terms is hardly affected by this fundamental arbitrariness. However, individual terms can exhibit quite a drastic change for certain proteins.

**Figure 4:**
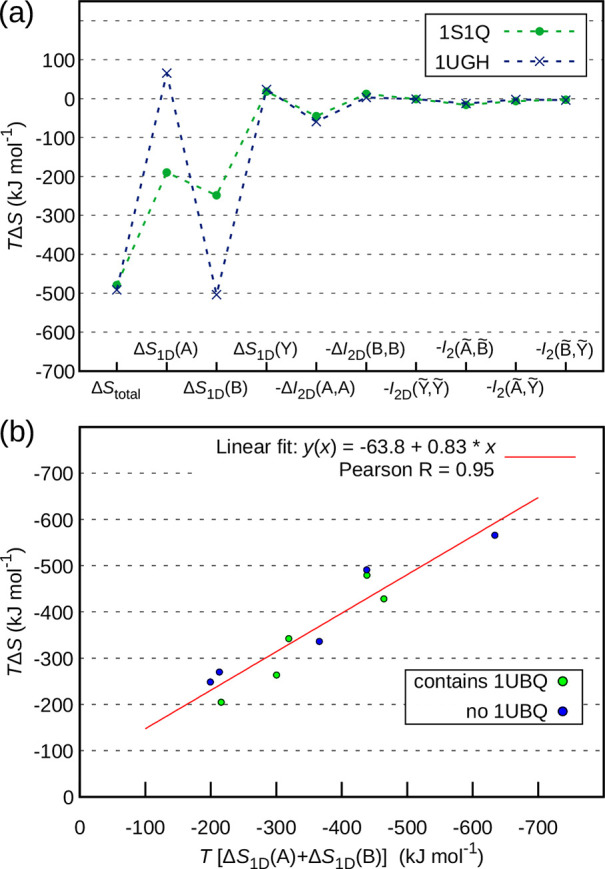
Relationship between different configurational entropy contributions. (a) Footprint of configurational entropy contributions for two specific complexes demonstrating the nonpredictable relationship between different terms. (b) The sum of the leading internal uncoupled terms is an excellent linear predictor of the total entropy change.

**Figure 5:**
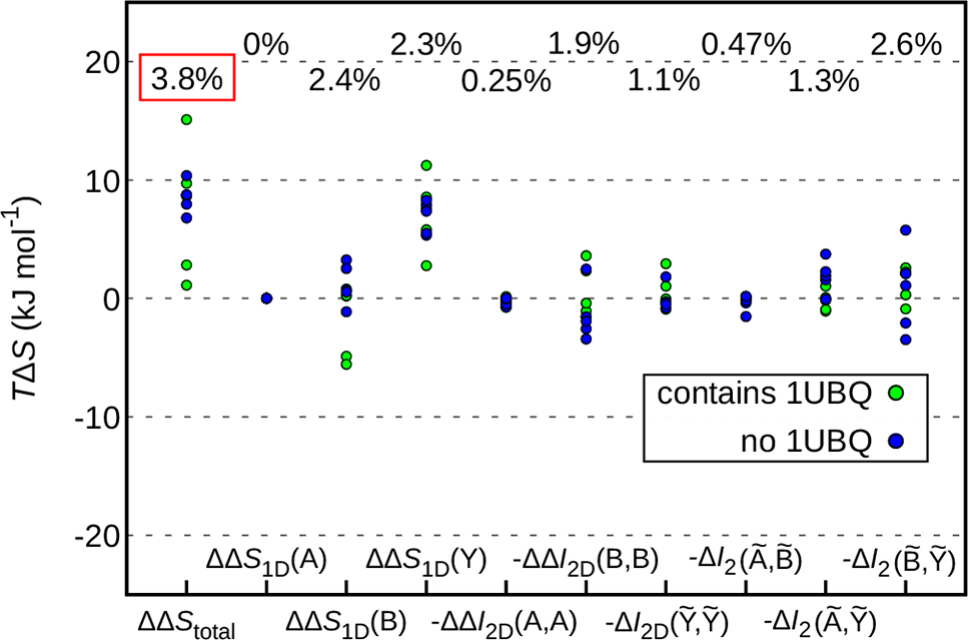
Effect of the choice of coordinate system on the importance of the entropy contributions. There is a largely arbitrary choice of root atoms to define the BAT coordinate system. Figure 3a shows values where the root atoms for each complex have been chosen (out of five sets of root atoms) in order to minimize Δ*S*_1D_(*Y*). The current graph shows the changes relative to this minimum choice when root atoms are chosen in order to maximize Δ*S*_1D_(*Y*). The percentage values describe the spans relative to the total entropy change in Figure 3a. Green points denote complexes involving ubiquitin (Figure 1a), while blue points denote complexes not involving ubiquitin (Figure 1b).

Generally, the footprint of a given molecule does not follow a readily discernible pattern, which is illustrated in the case of the 1S1Q and 1UGH complexes in Figure 4a (see Figure 1 and Table 1 for further details). While the two complexes exhibit almost the same total entropy change Δ*S*_total_, the contribution of the leading uncoupled terms Δ*S*_1D_ is vastly different. For 1S1Q, the two binding partners contribute similarly when it comes to Δ*S*_1D_. In 1UGH, however, a small Δ*S*_1D_ contribution of the larger binding partner 1AKZ is accompanied by a large Δ*S*_1D_ contribution of the smaller binding partner 1UGI. Surprisingly, however, the rest of the terms are virtually the same, which is noteworthy especially when it comes to the internal coupling terms. Remarkably, however, the sum of the internal uncoupled terms Δ*S*_1D_(*A*) + Δ*S*_1D_(*B*) exhibits an excellent linear correlation with the total entropy change for both the ubiquitin-containing and the non-ubiquitin-containing complexes (Figure 4b). This fact provides fundamental support for the recently developed NMR-based methods for measuring the configurational entropy change of protein interactions,^4,5,46,47^ which critically rely on such linear behavior. Nevertheless, although the external as well as the coupling terms obviously average out to a constant fraction rather well, given the ranges in Figure 3a, a customized recalibration for the system of interest (as done by the NMR methods), may likely be required for improved accuracy.

## 4 Conclusions

In summary, we have presented here a comprehensive theoretical framework for analyzing different contributions to configurational entropy change over internal and external degrees of freedom. Moreover, we have provided a quantitative assessment of the individual contributions to configurational entropy change in the case of a large set of MD simulations of biomolecular binding processes. While the analytical parts of our study are exact, the latter analysis was subject to different sources of uncertainty, including force field errors and convergence issues, and its results should be treated as such. We hope that these efforts will help to complete the theoretical foundation used for treating the configurational entropy in biomolecular systems. With recent methodological advances on both experimental and computational fronts, it is our firm conviction that such a foundation will be instrumental in numerous fundamental and applied contexts alike.

## 5 Methods

MD simulations were performed as described previously^36−38^ using the GROMACS 4.0.7 simulation package,^48,49^ the GROMOS 45A3 force field,^50^ and the SPC water model.^51^ Proteins were placed in water boxes, together with the necessary number of sodium or chloride counterions to reach neutrality, and subjected to energy minimization, followed by heating to 300 K for 100 ps and subsequent unconstrained MD simulations. The length of each MD trajectory was 1 μs, with the first 200 ns treated as an equilibration period and the remaining 800 ns analyzed. Simulations were carried out with a time step of 2 fs using 3D periodic boundary conditions, in the isothermal–isobaric (*NPT*) ensemble with an isotropic pressure of 1 bar and a constant temperature of 300 K, while system coordinates were output every 1 ps. The pressure and the temperature were controlled using the Berendsen thermostat and barostat^52^ with 1.0 and 0.1 ps relaxation parameters, respectively, and a compressibility of 4.5 × 10^–5^ bar^–1^ for the barostat. Bond lengths were constrained using LINCS.^53^ The van der Waals interactions were treated using a cutoff of 14 Å. Electrostatic interactions were evaluated using the reaction-field method,^54^ with a direct sum cutoff of 14 Å and relative permittivity of 61. For the complex 1YD8, due to the lack of a separate structure, the ubiquitin binding partner (human GGA3 GAT domain) was extracted from the PDB structure of the complex and equilibrated for an additional 500 ns. The PARENT^36^ program suite, a configurational entropy package in parallel architecture, was used for entropy calculations by applying the MIST approximation.^10,35^ For sampling probability densities, 50 bins were used in one-dimensional cases and 50 × 50 = 2500 in two-dimensional cases.

## Supporting Information Available

Convergence analysis from shuffled trajectories of the same three representative complexes as in the main article (PDF)
