# Supplementary material for: Configurational Entropy Components and Their Contribution to Biomolecular Complex Formation
Source: J Chem Theory Comput. 2019 May 1;15(6):3844–53. doi: 10.1021/acs.jctc.8b01254 (PMC9251725; doi:10.1021/acs.jctc.8b01254)
Supplement: Supplementary file 1 — ct8b01254_si_001.pdf [file ct8b01254_si_001.pdf]

Supplementary information:

Configurational Entropy Components and Their Contribution to Biomolecular Complex Formation

Markus Fleck[†,‡] and Bojan Zagrovic[*,†]

†*University of Vienna, Max F. Perutz Laboratories, Department of Structural and Computational Biology, Campus Vienna Biocenter 5, Vienna 1030, Austria.*

‡*University of Vienna, Faculty of Chemistry, Department of Computational Biological Chemistry, Währinger Straße 17, Vienna 1090, Austria*

E-mail: bojan.zagrovic@univie.ac.at

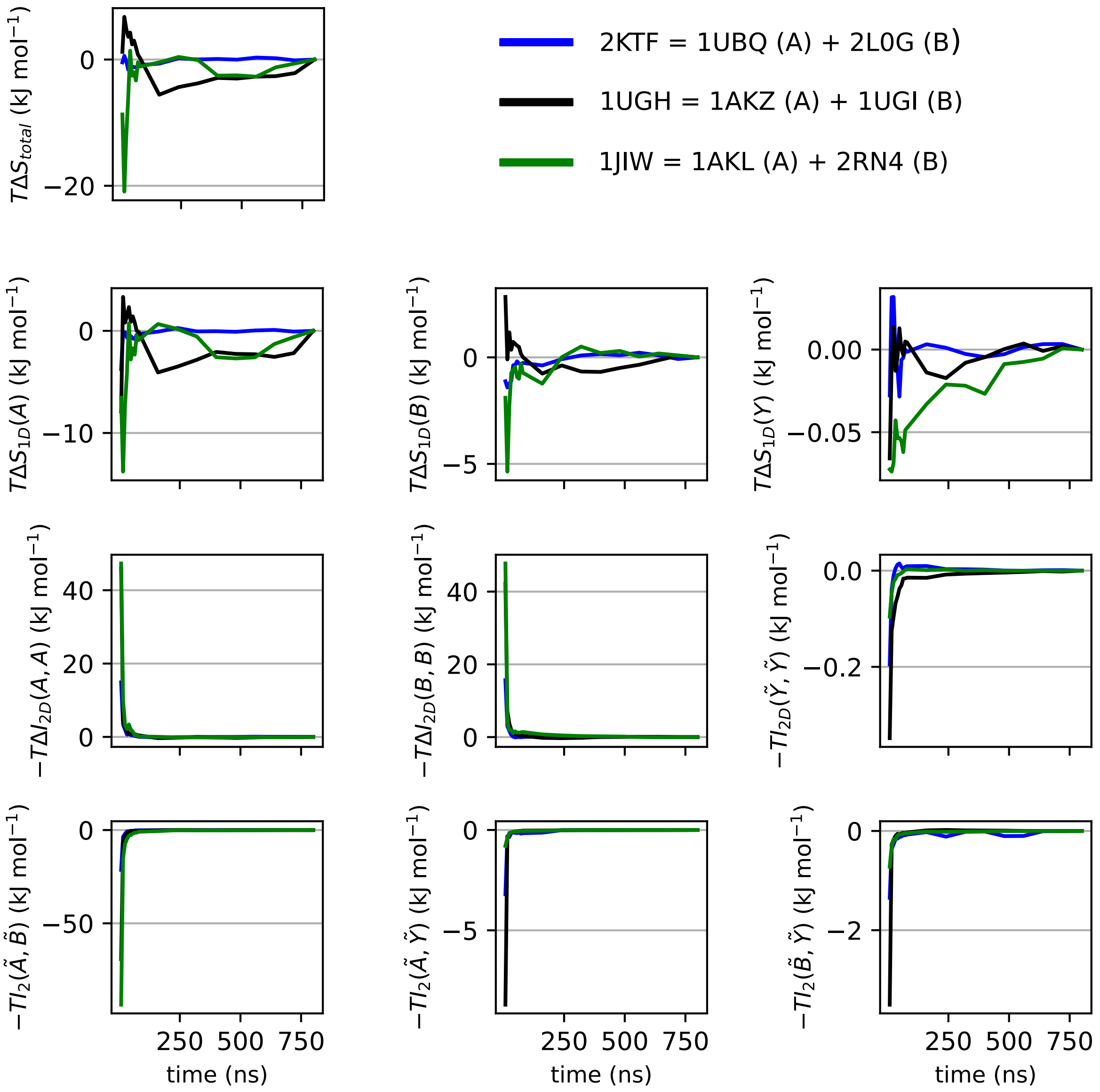

Figure 1: Convergence analysis of three representative complexes from shuffled trajectories. The analysis here is the same as in Figure 2 of the main article, but with the frames in the original trajectories shuffled randomly. By removing time ordering, this analysis captures convergence as a function of ensemble size (frames) as opposed to simulation time. Because the convergence with ensemble size is considerably more rapid, the first 80 ns have been fine-grained to 8 ns spacing of data points. The resolution of the trajectories is 1 ps.
